# Coronary Heart Disease and Cortical Thickness, Gray Matter and White Matter Lesion Volumes on MRI

**DOI:** 10.1371/journal.pone.0109250

**Published:** 2014-10-10

**Authors:** Miika Vuorinen, Soheil Damangir, Eini Niskanen, Julia Miralbell, Minna Rusanen, Gabriela Spulber, Hilkka Soininen, Miia Kivipelto, Alina Solomon

**Affiliations:** 1 Department of Neurology, School of Medicine, University of Eastern Finland, Kuopio, Finland; 2 Division of Clinical Geriatrics, NVS, Karolinska Institute, Novum, Stockholm, Sweden; 3 Department of Applied Physics, University of Eastern Finland, Kuopio, Finland; 4 Department of Psychiatry and Clinical Psychobiology, University of Barcelona, Barcelona, Spain; 5 Department of Neurobiology, Care Sciences and Society, KI- Alzheimer Disease Research Center (KI-ADRC), Karolinska Institute, Stockholm, Sweden; Indiana University School of Medicine, United States of America

## Abstract

Coronary heart disease (CHD) has been linked with cognitive decline and dementia in several studies. CHD is strongly associated with blood pressure, but it is not clear how blood pressure levels or changes in blood pressure over time affect the relation between CHD and dementia-related pathology. The aim of this study was to investigate relations between CHD and cortical thickness, gray matter volume and white matter lesion (WML) volume on MRI, considering CHD duration and blood pressure levels from midlife to three decades later. The study population included 69 elderly at risk of dementia who participated in the Cardiovascular Risk Factors, Aging and Dementia (CAIDE) study. CAIDE participants were examined in midlife, re-examined 21 years later, and then after additionally 7 years (in total up to 30 years follow-up). MRIs from the second re-examination were used to calculate cortical thickness, gray matter and WML volume. CHD diagnoses were obtained from the Finnish Hospital Discharge Register. Linear regression analyses were adjusted for age, sex, follow-up time and scanner type, and additionally total intracranial volume in GM volume analyses. Adding diabetes, cholesterol or smoking to the models did not influence the results. CHD was associated with lower thickness in multiple regions, and lower total gray matter volume, particularly in people with longer disease duration (>10 years). Associations between CHD, cortical thickness and gray matter volume were strongest in people with CHD and hypertension in midlife, and those with CHD and declining blood pressure after midlife. No association was found between CHD and WML volumes. Based on these results, long-term CHD seems to have detrimental effects on brain gray matter tissue, and these effects are influenced by blood pressure levels and their changes over time.

## Introduction

The incidence of cardiovascular conditions such as coronary heart disease (CHD) increases with age [1]. Similarly, the incidence of dementia and Alzheimer's disease (AD) increases notably after the age of 65 years [2]. These two chronic conditions share several risk factors, and CHD has also been associated with dementia [3]. Asymptomatic CHD is a common co-morbidity in stroke and transient ischemic attacks (TIA) [4], and silent cerebral infarcts seem to be more prevalent in patients with symptomatic CHD compared to the general population [5]. CHD and coronary artery calcification (CAC; a marker of atherosclerotic burden) have been associated with increased prevalence of white matter lesions (WML) [6], [7] and gray matter (GM) changes [8]. However, not all studies have found such associations [5], [9]. Furthermore, CHD has been related to AD neuropathology (neuritic plaques [NP] and neurofibrillary tangles [NFT]) [10], [11].

Studies focusing on CHD and structural brain changes have so far been cross-sectional and have not included more detailed measurements of GM and WM changes. In addition, although CHD is strongly associated with blood pressure (BP), it is not clear how BP levels or long-term changes in BP affect the relation between CHD and dementia-related pathology. Hypertension in midlife and low BP in late-life have been related to dementia, and declining BP was associated with structural brain changes [12]–[14]. While hypertension increases the risk of CHD, people with CHD may have reduced cardiac output leading to low BP [13].

The aim of this study was to investigate relations between CHD and cortical thickness, GM volume and WML volume on MRI, considering CHD duration and BP levels from midlife to three decades later.

## Methods

### Subjects and study design

**Figure 1:**
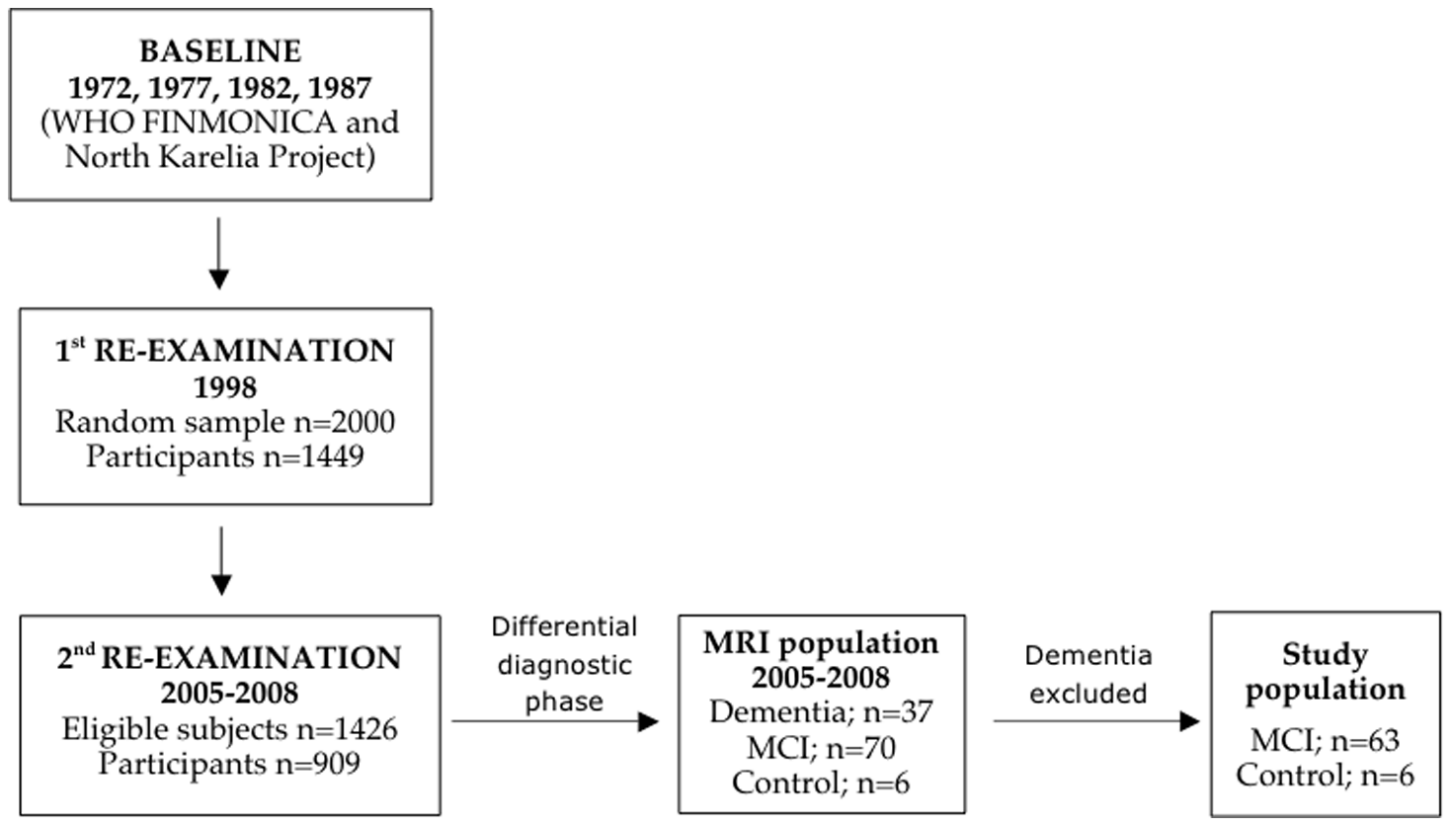
CAIDE study and formation of the MRI population.

The present study included 69 individuals who participated in the population-based Cardiovascular Risk Factors, Aging and Dementia (CAIDE) study. The CAIDE study and formation of the MRI population are summarized in **Figure 1**.

The CAIDE study has previously been described in detail [15]. Briefly, participants were examined in midlife (age≈50 years) within the framework of the North Karelia project and FINMONICA study in 1972, 1977, 1982, or 1987. In 1998, 2000 randomly selected survivors aged 65–79 years, and living in the Kuopio and Joensuu areas in Finland, were invited for a first re-examination. Altogether 1449 (72.6%) subjects participated. A second re-examination was conducted between 2005–2008. Of the initial 2000 persons, 1426 were still alive and living in the region, and 909 (63.7%) participated. In both re-examinations, cognition was assessed using a three-step protocol (screening, clinical phase, and differential diagnostic phase). At the first re-examination, participants with ≤24 points on Mini-Mental State Examination (MMSE) [16] at screening were referred for further evaluations. At the second re-examination, subjects with ≤24 points, or decline ≥3 points on MMSE, or <70% delayed recall in the CERAD word list [17], or with informant concerns about the participant's cognition were referred for further evaluations. At both re-examinations, the clinical phase included detailed medical and neuropsychological assessments, and the differential diagnostic phase consisted of brain imaging (MRI/CT), blood tests, and if needed cerebrospinal fluid (CSF) analysis. A review board including the study physician, neuropsychologist, a senior neuropsychologist and a senior neurologist ascertained the primary diagnosis based on all available information. Dementia was diagnosed according to the Diagnostic and Statistical Manual of Mental Disorders 4^th^ edition (DSM-IV) criteria [18], and AD according to the US National Institute of Neurologic and Communicative Disorders and Stroke-Alzheimer's Disease and Related Disorders Association (NINCDS-ADRDA) criteria [19]. A modified version of the Mayo Clinic Alzheimer's Disease Research Center criteria was used to identify mild cognitive impairment (MCI) [20].

At the second CAIDE re-examination, MRI scans were done only during the differential diagnostic phase for participants from Kuopio (113 subjects, of which 37 were diagnosed with dementia, 70 with MCI, and 6 were defined as controls). The imaged subjects had thus poorer performance in cognitive tests at screening compared to the rest of the CAIDE participants. Subjects with dementia were excluded from the present study, leaving a subsample of 69 elderly at risk of dementia (63 of them diagnosed with MCI) who also had MRI of adequate quality for cortical thickness measurements. The mean age (SD) of the 69 participants at baseline (midlife) was 49.9 (6.0) years, 70.5 (3.4) years at the first re-examination and 77.9 (3.5) years at the second re-examination. The mean follow-up time was (SD) was 20.6 (4.8) years between midlife and first re-examination, and 7.4 (0.3) years between the first and second re-examination. The CAIDE study was approved by the local ethics committee (University of Eastern Finland and Kuopio University Hospital, Kuopio, Finland), and written informed consent was obtained from all participants.

### CAIDE assessments and register CHD diagnoses

Survey methods used during the baseline (midlife) visit were standardized using international recommendations. They followed the WHO MONICA protocol in 1982 and 1987, and were comparable with the methods used in 1972 and 1977 [21]. Survey methods for re-examinations followed those for midlife visit in all aspects. Systolic blood pressure and diastolic blood pressure (SBP and DBP, respectively) were manually measured from subjects' right arm after seated down for five minutes. The APOE-genotypes were assessed from blood leucocytes using polymerase chain reaction and HhaI digestion [22].

CHD diagnoses were obtained from the Finnish Hospital Discharge Register (HDR) using ICD codes for ischemic heart diseases: 410-414 (ICD-8); 410-414 (ICD-9); I20-I25 (ICD-10). HDR includes information on in-patient stays in public hospitals (but not out-patient clinics, health centers or private hospitals) starting from 1969. The identification of persons in the register is based on the unique identification code given to every resident in Finland. The CHD diagnoses in HDR were previously shown to have overall sensitivity and positive predictive value above 80% [23].

### MRI acquisition

T1-weighted images were collected using a three-dimensional magnetization prepared rapid acquisition gradient echo (3D-MPRAGE) sequence on two different 1.5T MR scanners (Siemens Magnetom Vision or Siemens Avanto). The imaging parameters were: Magnetom Vision (repetition time [TR] = 9.7 ms, echo time [TE] = 4.0 ms, inversion time [TI] = 300 ms, flip angle [FA] = 12°, slice thickness = 1.5–2.0 mm, matrix 256×256, number of slices = 128 or 148) and Avanto (TR = 1900 ms, TE = 3.93 ms, TI = 1100 ms, FA = 15°, slice thickness = 1.0–1.5 mm, matrix 384/448×512, number of slices = 160). In addition, Fluid-Attenuated Inversion Recovery (FLAIR) images were collected as a part of imaging protocol (TR = 9000 ms, TE = 119 ms, TI = 2200 ms, slice thickness = 5 mm, flip angle = 180°, matrix = 512×168). All images were visually checked by an experienced neuroradiologist to confirm that they were free of artefacts and clinically significant brain conditions such as tumors, major post-stroke lesions or normal pressure hydrocephalus.

### Image analysis

Cortical thickness and brain tissues volume data were analysed using algorithms developed at McConnell Brain Imaging Centre, Montreal Neurological Institute, McGill University, Montreal, Canada (http://www.bic.mni.mcgill.ca/). Initially, individual native MRIs were registered into standardized stereotaxic space using the ICBM 152 template and corrected for intensity non-uniformity using the N3 algorithm. The N3 algorithm is a fully automated technique which maximizes the entropy of the intensity histogram and can be applied to any pulse sequences, field strength or MR scanner. Subsequently, the images were segmented into GM, WM and CSF using an artificial neural network classifier termed INSECT (Intensity-Normalized Stereotaxic Environment for Classification of Tissues) [24]. 3D brain mask was calculated to remove extra-cerebral voxels and partial volume effect (PVE) was estimated. Surfaces between GM and WM (WM surface = WMS) as well as GM and CSF (GM surface = GMS) were defined using Constrained Laplacian-based Automated Segmentation with Proximities (CLASP) algorithm [25]. Each polygon mesh surface consisted of 81 920 polygons and 40 962 nodes per hemisphere. Cortical thickness was defined as the distance between each vertex on WMS and its counterpart/linked vertex on GMS. Thickness calculations were performed in native space and thereafter transformed back to standardized space to enable group analysis. In the final step, cortical thickness maps were smoothed using 20 mm full width at half maximum diffusion kernel to increase the signal-to-noise ratio and to have more normally distributed data. Finally, the outcome of the pipeline was inspected visually to ascertain the quality of the surface estimation.

WML volumes were calculated using CASCADE, an automatic pipeline developed at Karolinska Institute, Stockholm, Sweden [26] (http://ki.se/en/nvs/cascade). Two MRI sequences (T1-weighted and FLAIR) were used as input for the image analysis. Pre-processing steps included affine registration of T1- to FLAIR-images; followed by brain extraction [27]. Manual quality control was performed to inspect the brain extraction quality. Then brain tissues segmentation [28] and histogram matching was carried out for both sequences. In the main classification, each voxel was classified as WML or normal based on the intensities of neighboring voxels on T1- and FLAIR-images. Voxels classified as normal were pruned away from cascade while the rest proceeded to the next step. Finally after multiple classification steps, only voxels classified as WML were left. Next morphological and spatial filtering was done to remove WML detections that were too small or in unlike spatial locations (e.g. in CSF). Finally all detections passed through boundary refining and the final output consisted of volumes and masks of the WML. By using this novel cascade method, high sensitivity (90%) and specificity (99.5%) was achieved when compared to manual delineation of WML [26].

### Statistical analysis

For cortical thickness, the statistical analyses were performed using in-house written Matlab scripts (Matlab R2008a, Mathworks Inc., Natick, Mass., USA). The areas where the cortical thickness was significantly different between subjects with or without CHD was defined using parametric t-tests performed for each vertex. The results were corrected for multiple comparisons using the false discovery rate (FDR) technique [29] (p<0.05). The minimum size for a region to be selected for more detailed analyses was 100 adjacent nodes that all passed the FDR-threshold. The mean absolute cortical thickness of these regions was calculated and exported to SPSS 19.0 (SPSS Inc., Chicago, IL, USA). Linear regression analyses were performed in SPSS to investigate the possible effects of CHD, or CHD and BP on the mean regional cortical thickness, WML volume (log-transformed to normalize distribution) and GM volume. All analyses were adjusted for age, sex, follow-up time and scanner type. Diabetes, cholesterol and smoking were added separately to this model, but this had no effect on the results and they were dropped out from the final analyses to avoid overadjustments. GM volume analyses were also adjusted for total intracranial volume (TIV). Results are presented as standardized β-coefficients (p-values). Several definitions were used for CHD: all CHD diagnosed until the first CAIDE re-examination; all CHD diagnosed until the second re-examination; and CHD with shorter/longer duration. Duration of CHD was calculated as number of years between the first date of diagnosis and date of second CAIDE re-examination, and categorized into two groups (11 subjects with CHD duration ≤10 years, and 15 subjects with CHD duration>10 years). To investigate the combined effects of CHD and BP on MRI outcomes at the second CAIDE re-examination, we used CHD diagnosed until the first CAIDE re-examination, midlife BP, and changes in BP between midlife and second re-examination. Midlife hypertension was defined as SBP≥160 and/or DBP≥95 due to the generally high BP levels in Eastern Finland at the time of the midlife examination ([15], Table 1), and in order to form groups of reasonable size for the analyses.

**Table 1:** Population characteristics according to history of CHD diagnosed until the first CAIDE re-examination.

Characteristics	All (n = 69)	No CHD (n = 50)	CHD (n = 19)	p-value
**Age in midlife** (baseline), years	49.87 (6.01)	49.19 (6.21)	51.66 (5.18)	0.13
**Age at 1^st^ re-examination** a, years	70.51 (3.43)	70.37 (3.41)	70.89 (3.55)	0.58
**Age at 2^nd^ re-examination**, years	77.95 (3.49)	77.78 (3.46)	78.40 (3.63)	0.51
**Sex (men)**, n (%)	27 (39.1)	16 (32.0)	11 (57.9)	0.05
**Follow-up time**, years	28.08 (4.72)	28.59 (4.77)	26.75 (4.44)	0.15
**Education**, years	7.76 (2.59)	7.51 (2.46)	8.44 (2.90)	0.19
**SBP in midlife**, mmHg	148.86 (24.79)	146.72 (26.72)	154.47 (18.23)	0.25
**SBP at 1^st^ re-examination** a, mmHg	158.61 (23.87)	162.79 (21.04)	147.37 (27.89)	**0.03**
**SBP at 2^nd^ re-examination**, mmHg	148.12 (21.54)	151.78 (22.31)	138.47 (16.22)	**0.02**
**DBP in midlife**, mmHg	90.55 (11.27)	89.50 (11.63)	93.32 (10.02)	0.21
**DBP at 1^st^ re-examination** a, mmHg	85.15 (10.78)	86.60 (10.94)	81.25 (9.60)	0.09
**DBP at 2^nd^ re-examination**, mmHg	75.75 (11.20)	77.36 (10.98)	71.53 (10.95)	**0.05**
**APOE ε4 carrier** b, n (%)	22 (38.6)	17 (40.5)	5 (33.3)	0.65
**Total GM volume**, ml	615.38 (71.16)	617.21 (64.92)	610.54 (87.30)	0.73
**Total intracranial volume**, ml	1339.91 (143.23)	1326.24 (137.20)	1375.89 (156.12)	0.20
**Total WML volume**, ml	33.02 (29.72)	31.79 (26.58)	36.23 (37.41)	0.81
**Region of interest, mean cortical thickness** c, mm				
Left anterior insular cortex	4.29 (0.29)	4.37 (0.24)	4.06 (0.28)	**<0.01**
Right anterior insular cortex	4.13 (0.30)	4.21 (0.24)	3.90 (0.32)	**<0.01**
Left angular gyrus	3.10 (0.29)	3.18 (0.22)	2.90 (0.35)	**<0.01**
Right angular gyrus	3.12 (0.25)	3.18 (0.20)	2.97 (0.32)	**<0.01**
Left fusiform gyrus	3.56 (0.22)	3.62 (0.17)	3.42 (0.28)	**<0.01**
Right fusiform gyrus	3.36 (0.22)	3.43 (0.18)	3.20 (0.23)	**<0.01**
Left anterior prefrontal cortex	3.60 (0.27)	3.67 (0.23)	3.42 (0.29)	**<0.01**
Right anterior prefrontal cortex	3.05 (0.29)	3.11 (0.25)	2.88 (0.33)	**<0.01**
Left superior parietal gyrus	3.04 (0.26)	3.10 (0.23)	2.88 (0.29)	**<0.01**
Left superior temporal gyrus	3.14 (0.24)	3.21 (0.16)	2.97 (0.33)	**<0.01**
Right posterior middle frontal gyrus	2.99 (0.26)	3.06 (0.18)	2.82 (0.36)	**<0.01**
Right orbitofrontal area	3.70 (0.31)	3.77 (0.24)	3.50 (0.39)	**<0.01**
Right precentral gyrus	2.44 (0.24)	2.49 (0.22)	2.29 (0.24)	**<0.01**
Right inferior frontal gyrus	2.95 (0.27)	3.02 (0.20)	2.78 (0.34)	**<0.01**

Values are means (SD) for continuous variables and numbers (%) for categorical variables. P-value presents the statistical significance of characteristic differences between no CHD and CHD groups. P-values <0.05 are bold.

a 10 subjects did not participate at the first CAIDE re-examination.

b APOE genotype information missing for 12 subjects.

c Mean cortical thickness (mm) was calculated for all statistically significant (p-value <0.05, FDR-corrected) areas that included more than 100 adjacent nodes.

Population characteristics were analyzed with SPSS software using cross-tabulation and χ^2^ for categorical variables and independent samples t-test for continuous variables. The level of significance was set to p<0.05 in all analyses.

## Results

The 69 participants included in the MRI population were not significantly different from the rest of the individuals in the original CAIDE population with respect to age at baseline (p = 0.3), gender (p = 0.8), education (p = 0.2), midlife SBP (p = 0.2) or DBP (p = 0.6), midlife total cholesterol (p = 0.09), or CHD diagnosed during the study (p = 0.4).

Only three of the 69 MRI participants had CHD at baseline (midlife), 19 at the time of the first CAIDE re-examination, and 26 at the second re-examination. Population characteristics according to history of CHD diagnosed until the first re-examination are shown in **Table 1**. Men tended to have CHD more often compared to women. Midlife SBP and DBP were not significantly different between people with and without CHD, but participants with CHD had lower SBP and DBP at both re-examinations.

**Figure 2:**
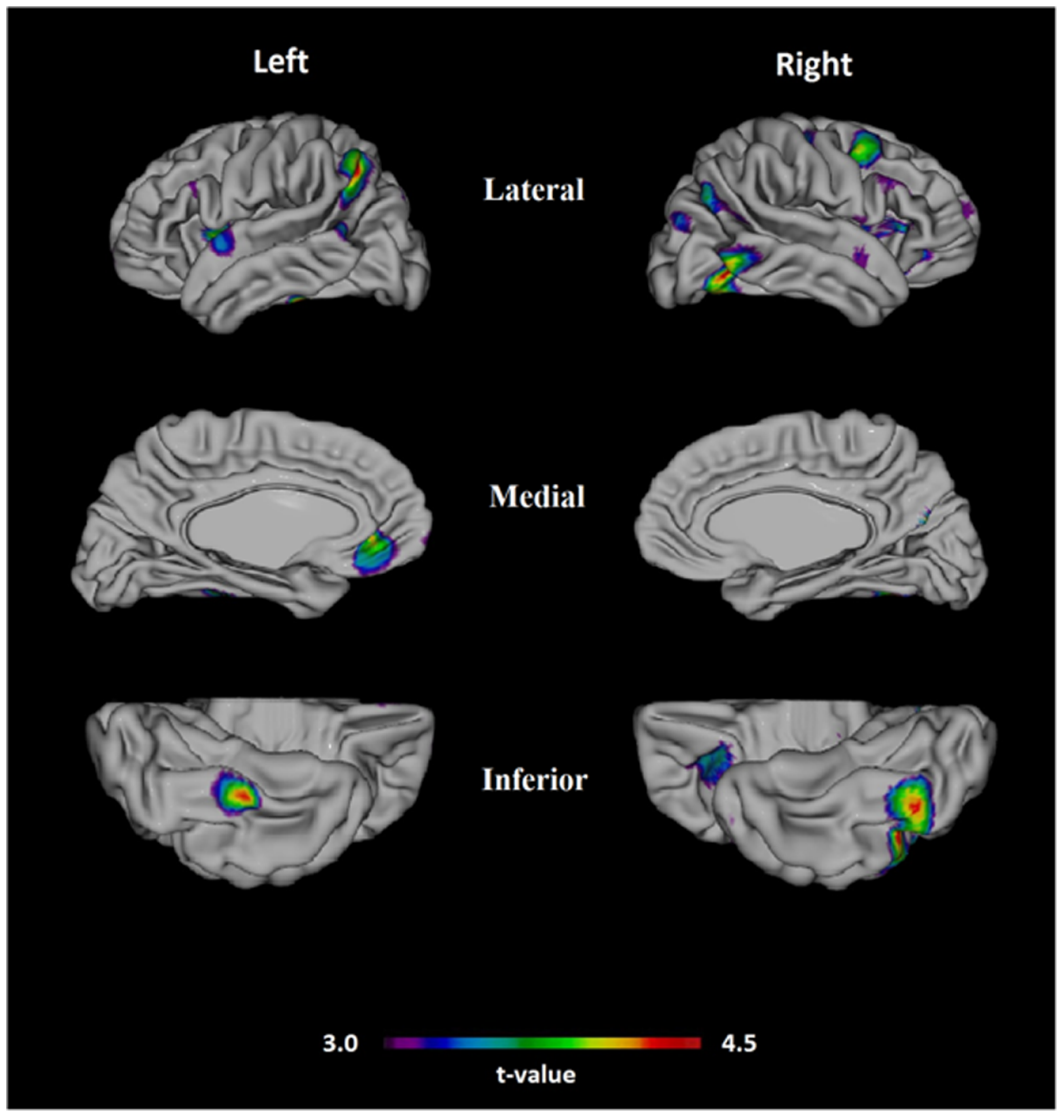
Statistical difference (t-value) maps of cortical thickness between CHD and control groups. Significant and FDR corrected (p<0.05) differences in cortical thickness between groups are displayed as color-labeled t-values on the surface of a standardized brain (warmer colors indicate thinner cortex). Analyses are adjusted for age, sex, follow-up time and scanner type.

Compared to subjects without CHD, those with CHD diagnosed until the first CAIDE re-examination had thinner cerebral cortex in multiple areas on MRI at the second re-examination (**Figure 2**). Thinner cortex was observed bilaterally in the anterior insular cortex, fusiform gyrus, anterior prefrontal cortex and angular gyrus; and additionally in the left superior termporal gyrus, left superior parietal gyrus, right orbitofrontal area, right precentral gyrus, right posterior middle frontal gyrus and right inferior frontal gyrus. When analyses were additionally adjusted for midlife SBP, only three areas showed significant results: right posterior middle frontal gyrus, inferior temporal gyrus, and fusiform gyrus. When all CHD diagnoses until the second re-examination were taken into account, a similar but non-significant relation was found between cortical thickness and CHD (results not shown). Stratification of analyses according to APOE genotype did not change the results.

People with CHD diagnosed until the first or the second CAIDE re-examination had lower GM volume at the second re-examination: standardized β-coefficients (p-values) were -0.150 (0.018) and -0.129 (0.041), respectively. No significant associations between CHD diagnosed until the first or the second CAIDE re-examination and WML volume were found: standardized β-coefficients (p-values) were -0.046 (0.719), and 0.054 (0.670).

When duration of CHD was taken into account, the associations with thinner cortex and smaller GM volume were consistently significant in the group with longer disease duration (**Table 2**). CHD was associated with lower cortical thickness in right anterior insular cortex and left fusiform gyrus irrespective of disease duration.

**Table 2:** Associations between CHD and cortical thickness, GM volume and WML volume according to the duration of CHD.

MRI measurements	CHD duration ≤10 years	CHD duration>10 years
**Mean cortical thickness**		
• Left anterior insular cortex	−0.170 (0.139)	**−0.499 (<0.001)**
• Right anterior insular cortex	**−0.311 (0.007)**	**−0.452 (<0.001)**
• Left angular gyrus	−0.075 (0.512)	**−0.464 (<0.001)**
• Right angular gyrus	−0.165 (0.138)	**−0.417 (<0.001)**
• Left fusiform gyrus	**−0.273 (0.028)**	**−0.385 (0.002)**
• Right fusiform gyrus	−0.204 (0.094)	**−0.458 (<0.001)**
• Left anterior prefrontal cortex	−0.098 (0.407)	**−0.401 (0.001)**
• Right anterior prefrontal cortex	−0.185 (0.128)	**−0.379 (0.002)**
• Left superior parietal gyrus	−0.163 (0.174)	**−0.384 (0.002)**
• Left superior temporal gyrus	−0.153 (0.216)	**−0.340 (0.007)**
• Right posterior middle frontal gyrus	−0.193 (0.075)	**−0.481 (<0.001)**
• Right orbitofrontal area	−0.046 (0.714)	**−0.338 (0.008)**
• Right precentral gyrus	−0.167 (0.160)	**0.435 (<0.001)**
• Right inferior frontal gyrus	−0.172 (0.134)	**−0.413 (0.001)**
**Total GM volume**	0.002 (0.972)	**−0.191 (0.002)**
**Total WML volume**	0.207 (0.100)	−0.090 (0.470)

Values are standardized β-coefficients (p-values) from linear regression analyses. All analyses are adjusted for age, sex, follow-up time and scanner type. GM volume analyses are additionally adjusted for TIV. P-values <0.05 are bold.

**Table 3** shows the effects of midlife hypertension and changes in SBP from midlife to the second re-examination on the associations between CHD and MRI measurements. The strongest relations between CHD diagnosed until the first re-examination and thinner cortex or lower GM volume at the second re-examination were found in participants with both CHD and midlife hypertension. Having CHD without midlife hypertension was also related to decreased cortical thickness in some regions, but the associations were weaker. In participants without CHD but with midlife hypertension, total WML volume tended to be higher compared to participants with no CHD and no midlife hypertension.

**Table 3:** Associations of CHD (diagnosed until the first CAIDE re-examination) and BP with regional cortical thickness, GM and WML volumes.

MRI measurements	No CHD	No CHD	CHD	CHD	No CHD	No CHD	CHD	CHD
	No midlife hypertension (N = 34)	Midlife hypertension (N = 16)	No midlife hypertension (N = 9)	Midlife hypertension (N = 10)	Stable/increasing SBP (N = 31)	Declining SBP (N = 19)	Stable/increasing SBP (N = 7)	Declining SBP (N = 12)
**Mean cortical thickness**								
Left anterior insular cortex	Ref	0.01 (0.96)	−0.17 (0.15)	**−0.54 (<0.01)**	Ref	0.03 (0.77)	−0.18 (0.13)	**−0.48 (<0.01)**
Right anterior insular cortex	Ref	−0.13 (0.21)	−0.14 (0.20)	**−0.61 (<0.01)**	Ref	−0.12 (0.27)	−0.14 (0.22)	**−0.55 (<0.01)**
Left angular gyrus	Ref	−0.13 (0.27)	*−0.23 (0.05)*	**−0.50 (<0.01)**	Ref	−0.04 (0.74)	**−0.31 (0.01)**	**−0.39 (<0.01)**
Right angular gyrus	Ref	0.04 (0.68)	−0.10 (0.35)	**−0.47 (<0.01)**	Ref	−0.05 (0.67)	−0.17 (0.16)	**−0.43 (<0.01)**
Left fusiform gyrus	Ref	0.02 (0.88)	−0.14 (0.23)	**−0.52 (<0.01)**	Ref	−0.04 (0.73)	**−0.30 (0.02)**	**−0.41 (<0.01)**
Right fusiform gyrus	Ref	−0.04 (0.70)	*−0.20 (0.08)*	**−0.62 (<0.01)**	Ref	0.01 (0.91)	*−0.22 (0.07)*	**−0.54 (<0.01)**
Left anterior prefrontal cortex	Ref	−0.06 (0.62)	**−0.24 (0.04)**	**−0.46 (<0.01)**	Ref	−0.02 (0.83)	*−0.20 (0.098)*	**−0.45 (<0.01)**
Right anterior prefrontal cortex	Ref	−0.06 (0.59)	−0.12 (0.31)	**−0.48 (<0.01)**	Ref	−0.08 (0.51)	*−0.24 (0.07)*	**−0.38 (<0.01**
Left superior parietal gyrus	Ref	0.05 (0.66)	−0.16 (0.19)	**−0.42 (<0.01)**	Ref	−0.02 (0.87)	−0.15 (0.21)	**−0.42 (<0.01)**
Left superior temporal gyrus	Ref	−0.05 (0.66)	−0.14 (0.24)	**−0.52 (<0.01)**	Ref	−0.10 (0.40)	**−0.27 (0.03)**	**−0.42 (<0.01)**
Right posterior middle frontal gyrus	Ref	−0.07 (0.52)	−0.06 (0.54)	**−0.59 (<0.01)**	Ref	*−0.21 (0.05)*	*−0.21 (0.06)*	**−0.50 (<0.01)**
Right orbitofrontal area	Ref	−0.03 (0.80)	−0.07 (0.54)	**−0.58 (<0.01)**	Ref	−0.07 (0.57)	−0.12 (0.34)	**−0.51 (<0.01)**
Right precentral gyrus	Ref	−0.03 (0.80)	−0.09 (0.42)	**−0.52 (<0.01)**	Ref	−0.13 (0.30)	−0.17 (0.17)	**−0.46 (<0.01)**
Right inferior frontal gyrus	Ref	−0.10 (0.34)	−0.07 (0.51)	**−0.54 (<0.01)**	Ref	−0.18 (0.12)	*−0.22 (0.07)*	**−0.43 (<0.01)**
**Total GM volume**	Ref	0.07 (0.29)	−0.03 (0.64)	**−0.16 (0.01)**	Ref	−0.04 (0.51)	−0.09 (0.16)	**−0.15 (0.02)**
**Total WML volume**	Ref	*0.24 (0.07)*	−0.02 (0.88)	0.06 (0.63)	Ref	0.13 (0.32)	−0.02 (0.90)	−0.01 (0.92)

Numbers are standardized beta-coefficients (p-value) from linear regression analyses. Analyses are adjusted for age, sex, follow-up time and scanner type. GM volume analyses are additionally adjusted for TIV.

P-values <0.05 are bold and trends (p-value <0.1) are italic.

Subjects with CHD and declining SBP between midlife and second re-examination had significantly thinner cortex and lower GM volume compared to subjects with no CHD and stable SBP levels. CHD was related to thinner cortex in some of the regions of interest even in participants with stable/increasing SBP, but the associations were weaker. A similar pattern of association was found for CHD and changes in DBP from midlife to late-life in relation to cortical thickness and GM volume in late-life (results not shown).

## Discussion

CHD diagnosed at least seven years before MRI scans was associated with decreased cortical thickness and lower total GM volume in a population-based sample of 69 elderly at risk of dementia. Similar but less strong associations were found when all CHD diagnoses until the time of the MRI scans were considered. The effects of CHD on cortical thickness and GM volume were most pronounced in people with a longer duration of CHD (>10 years).

The cortical areas related to CHD in the present study are involved in autonomic functions such as BP and heart rate control (insular cortex) [30], and in cognitive domains such as executive functioning (prefrontal cortex) [31], face and word recognition (fusiform gyrus) [32] and language skills (angular gyrus) [33]. The insular cortex is situated in the region of the end arterioles of the middle cerebral arteries, which makes it vulnerable to vascular disease [30]. In addition, the insular cortex has been shown to be thinner in subjects with AD, and both insular pathology and autonomic dysfunction are known to occur in AD [34]. Traditionally the fusiform gyrus has been related to face and word recognition [32], but previous studies have also linked it with amnestic mild cognitive impairment [35] and decreased MMSE score [36]. In our study, cortical thinning was also observed in the frontal regions in people with CHD. CHD is strongly related to hypertension, which is associated with both frontal gray and white matter changes [37], [38]. In a previous cross-sectional study, CHD was linked with medial frontal lobe volume decrease [39].

The association between CHD and lower total GM volume is in line with a previous study reporting detrimental effects of CHD on GM [8]. However, not all studies have found such effects [5], [9], and differences in results may be due to differences in MRI methods and populations. The CAIDE MRI subsample is a population of at-risk elderly with somewhat lower performance on cognitive tests, who may already have some vascular or AD-related neuropathology. CHD has been shown to increase the risk of NP and NFT accumulation in the brain, especially in APOE ε4 carriers [11]. Possible mechanisms behind this phenomenon could be cerebrovascular arterial atherosclerosis [40], and the effects of APOE on brain cholesterol metabolism [41]. We did not find any relation between CHD, the APOE ε4 allele and brain changes in this study, but this may be due the lack of statistical power.

Because CHD is strongly related to hypertension [12], and midlife hypertension and declining BP from midlife to late-life have been associated with lower GM volume, and thinner cortex [14], [42], we did additional analyses taking both CHD and BP into account. The strongest associations between CHD, thinner regional cortex and lower total GM volume were found in people with both CHD and midlife hypertension, and in people with both CHD and declining SBP or DBP after midlife. A more pronounced decline in BP has been described earlier in people who develop dementia later on [43], [44]. Midlife hypertension can increase the risk of cerebrovascular or Alzheimer-related brain changes, and it may also affect the brain areas involved in BP regulation (i.e. insular cortex), which may in turn lead to declining BP after midlife [14]. Maintaining adequate BP levels is essential in patients with CHD [45]. Declining BP (especially DBP) in CHD could be a marker of left ventricular dysfunction due to myocardial ischemia. Systemic hypoperfusion may lead to substantial cerebral hypoperfusion, exposing brain tissue to hypoxia [46]. Only four subjects with CHD had been diagnosed with heart failure in our study, and the effects of heart failure could not be assessed.

In the CAIDE MRI population, having CHD without midlife hypertension, or CHD with stable/increasing SBP/DBP after midlife was also associated with lower cortical thickness in some regions, suggesting that the effects of CHD on the brain may be partly independent of BP. However, more detailed analyses on the interplay between CHD and BP in relation to MRI findings were not possible due to the small sample size.

No significant differences in WML volume were found between people with and without CHD, although having midlife hypertension without CHD tended to relate to higher WML volume. Measures of artery calcification have been linked to WML in several studies [7], [47], but findings on CHD and WML are less consistent [5], [6], [8], [9].

The main strengths of this study are the population-based sample with long follow-up time starting already in midlife, detailed MRI measurements, and the use of CHD information from a validated national register. However, results need to be interpreted with caution due to the small sample size and limitations of statistical power. In addition, our findings may underestimate the effects of CHD due to selective survival and participation bias (i.e. people with more severe CHD were less likely to survive to older ages or to participate in the study), and because the register only records CHD cases severe enough to require hospitalization. No quantitative measures of ventricular function (e.g. ejection fraction) were available, so milder forms of heart failure could not be identified.

Two different scanners from the same manufacturer (Siemens) were used for acquiring MRIs which can be considered as a limitation. However, no significant differences in brain or WML volumes were noticed between scanners, in agreement with previous findings [48]. All analyses were adjusted for scanner type.

In conclusion, our results suggest that CHD (particularly with longer disease duration) is related to lower cortical thickness and GM volume, and this association is influenced by BP. People with CHD and midlife hypertension, and those with CHD and declining BP may be at greater risk of GM atrophy. Further studies will need to examine in more detail to what extent these associations are explained by neurodegenerative pathology, vascular mechanisms or a combination of both, depending on the interplay between CHD, BP and possibly other coexistent risk factors from midlife to old age.
